# The Influence of Solution Temperature on Fluid Deficit During Hysteroscopy

**DOI:** 10.1007/s43032-026-02151-2

**Published:** 2026-07-13

**Authors:** Aya Mohr-Sasson, Shannon Chang, Asha Bhalwal, Randa Jalloul, Mateo G. Leon, Olivia Dziadek, Kelsey Schmidt, Alvaro Montealegre

**Affiliations:** Advanced Minimally Invasive Gynecologic Surgery, Department of Obstetrics, Gynecology & Reproductive Sciences, McGovern Medical School at The University of Texas Health Science Center, 6431 Fannin, MSB 3.286, Houston, TX 77030 USA

**Keywords:** Hysteroscopy, Fluid temperature, Fluid deficit

## Abstract

**Abstract:**

Warm distension media have been introduced in office hysteroscopy to reduce procedure-related pain, yet concerns persist that vasodilation from warmed fluid may accelerate intravasation and increase fluid deficit. Evidence linking irrigation fluid temperature with intravasation risk is limited and largely theoretical. The aim of this study was to evaluate the association between the irrigation fluid temperature on the fluid deficit during hysteroscopic procedures. This is a randomized controlled study conducted at a medical center, including all women scheduled for operative hysteroscopy due to preoperative diagnoses of polyps, endometrial thickening, or uterine fibroids (FIGO classification: type 0, 1, or 2). Participants were randomized to receive irrigation fluid at either room temperature (24 °C) or body temperature (37 °C) and were blinded to allocation. The primary outcome was the time to reach a 250 mL fluid deficit. A total of 91 women met inclusion criteria, of whom 44(48%) were in the room temperature group and 47(52%) were in the body temperature group. Indications, age, BMI, device type, and tissue-shaver use were similar between groups. No significant difference was observed in the time to reach a 250 mL fluid deficit [155 (67–314) vs. 219 (83–444) seconds for the room temperature and body temperature groups, respectively; *p* = 0.25]. Exploratory analyses at higher fluid deficit thresholds similarly showed no significant differences between groups. A trend of a higher visual analog pain score (VAS) was reported by the women in the room temperature group compared to the warmed fluid group [4(0–6) vs. 1(0–6); *p* = 0.38]. Irrigation fluid temperature did not significantly impact fluid deficit during hysteroscopy. Warmed fluid may offer improved patient comfort without increasing intravasation risk.

**Synopsis:**

Warm distension fluid is used in office hysteroscopy to reduce pain, but it may limit procedure duration due to increased vascular expansion and fluid deficit. This study compared fluid deficits at 24 °C (room temperature) and 37 °C (body temperature) during hysteroscopy. Results showed no significant impact of fluid temperature on deficits, regardless of the procedure indication.

**Supplementary Information:**

The online version contains supplementary material available at 10.1007/s43032-026-02151-2.

## Introduction

Operative Hysteroscopy Intravascular Absorption (OHIA) syndrome is a significant complication of operative hysteroscopy, arising from excessive fluid absorption during the procedure [1]. Although rare, severe fluid overload can result in critical conditions requiring intensive care, such as dilutional hyponatremia, hypoosmolality, cerebral edema, hypokinetic circulation, and cardiovascular collapse [2].

During surgical resection, hydrostatic pressure typically exceeds intravascular pressures, particularly within the venous system, facilitating the intravasation of irrigation fluid from the surgical field into the intravascular space [3]. The greater the disparity between these pressures, the higher the flow rate into the bloodstream and the greater the risk of fluid overload. Furthermore, despite efforts to minimize surgical time and maintain low distension pressures, fluid overload can still occur unexpectedly and before the anticipated timeframe [2].

Preventive measures, such as low-pressure irrigation, can help reduce fluid absorption but do not completely eliminate the risk of this complication. Monitoring fluid absorption during surgery is essential for maintaining fluid balance in individual patients.

Reports examining the clinical relationship between irrigation fluid temperature and intravasation rates or the risk of fluid overload are scarce and primarily based on theoretical models [4]. Studies on transurethral prostatic resection (TUR) have reported benefits of cold irrigation fluids, including shorter procedure duration, improved surgical visualization, reduced fluid use, and decreased blood loss [5, 6]. However, the evidence regarding increased complication rates due to hypothermia from locally applied cold irrigation solutions is mixed, and such findings have not been specifically demonstrated in hysteroscopic procedures [6, 7].

The use of warm distension fluid in office hysteroscopy has recently been introduced to reduce pain severity [8]. A recent meta-analysis of five randomized controlled trials reported that warm saline was associated with a significant reduction in the visual analog scale (VAS) pain score during the procedure and higher patient satisfaction [9]. However, warm irrigation solutions may be limiting for longer procedures, as the expected expansion of the vascular system can lead to a higher fluid deficit in a shorter time.

This study aims to evaluate the impact of irrigation fluid temperature on fluid deficit during hysteroscopic procedures, comparing two temperature setups: 24 °C (room temperature) and 37 °C (body temperature). Based on previous studies, we hypothesize that the use of warmed fluid will result in a larger fluid deficit, while potentially reducing postoperative pain perception.

## Material and Methods

This is a prospective randomized controlled trial conducted at a single university affiliated tertiary medical center between July 2023 to July 2024. All women undergoing gynecologic operative hysteroscopic surgery with preoperative diagnosis of endometrial polyp, endometrial thickening or uterine fibroids with FIGO classification: type 0, 1 or 2, were invited to participate in the study. Women with known malignancy, contraindication for hysteroscopic surgery (such as active genital infection) or women unable to provide consent were excluded. Procedures performed with manual evaluation of fluid deficit or alternative irrigation solutions (Ringer’s lactate, 1.5% glycine, 5% dextrose) were also excluded. Following signing of informed consent, participants were randomized using a 1:1 block randomization scheme based on sequential enrollment order. Women with odd-numbered enrollment positions were assigned to receive hysteroscopic distension media at 24 °C (room temperature), while those with even-numbered positions received media at 37 °C (body temperature). Participants scheduled for hysteroscopic myomectomy were randomized separately using the same allocation method to ensure balanced distribution within this subgroup. Allocation concealment was maintained, and all participants were blinded to the temperature assignment of the distension media. Blinding of the surgical team was not feasible, as the use of warming devices and handling of heated distension media are inherent to the procedure and readily apparent in the operating room environment.

All surgeries were performed by a consistent specialized surgical team in benign gynecological minimally invasive surgery that includes 6 surgeons. As all procedures were performed under general anesthesia, no preoperative analgesia was administered. A TruClear™ mechanical hysteroscope, with a fluid collecting system was used in all of the procedures using 0.9% Normal Saline solution as the expanding media. The TruClear™ mechanical hysteroscope system includes two main models: the Mini TruClear™ with an outer diameter of approximately 4.5 mm, and the Dense TruClear™ measuring about 6.5 mm in diameter. Both models utilize a mechanical cutting system featuring a rotating and oscillating blade that simultaneously cuts and aspirates tissue, enabling continuous fluid flow and improved visualization during hysteroscopic procedures. Each scope also offers interchangeable options for soft or dense tissue shaving, allowing customization based on the consistency of the targeted intrauterine pathology. The choice between the mini or plus TruClear™ hysteroscope was determined by the pathology and the surgeon's preference. Similarly, when needed, the selection of mini or dense tissue shavers was guided by the pathology and the surgeon's discretion.

An additional advantage of this system is its integrated fluid management capability, which allows real-time assessment and monitoring of fluid deficit, thereby enhancing procedural safety. The outflow of hysteroscopic fluid was monitored using the integrated closed suction and collection system of the TruClear™ mechanical hysteroscope, which includes a dedicated drain line that captures and measures fluid outflow in real-time.

Surgery was started with vaginoscopy without cervical dilatation, when possible, followed by entry to the cervical canal and uterine cavity using hydrodistension. Vaginoscopy provides a minimally invasive approach for uterine access that minimizes cervical trauma and mechanical manipulation, advantages that persist even during operative procedures under anesthesia. By eliminating the need for speculum insertion and cervical dilation, this technique reduces intraoperative tissue disruption, which may enhance surgical outcomes and expedite postoperative recovery. Furthermore, the adoption of vaginoscopy permits more precise differentiation of postoperative pain etiology, enabling clinicians to attribute pain specifically to intrauterine interventions rather than confounding discomfort associated with vaginal or cervical instrumentation.

The distention medium utilized in this study was isotonic 0.9% normal saline, selected for its safety profile and widespread use in hysteroscopic procedures. For the warmed fluid group, the saline solution was maintained at a controlled temperature by storing it in a calibrated incubator set to 37°C. To preserve the temperature integrity of the irrigation fluid, it was transported to the operating room immediately prior to the procedure’s timeout and only opened after anesthesia induction and patient draping were completed. This protocol was implemented to minimize thermal loss and maintain the irrigation fluid at or near physiological temperature throughout the hysteroscopic procedure. Given the short duration of the procedures, no intraoperative temperature monitoring was performed. The distention medium utilized in this study was isotonic 0.9% normal saline, selected for its safety profile and widespread use in hysteroscopic procedures. For the warmed fluid group, the saline solution was maintained at a controlled temperature by storing it in a calibrated incubator set to 37°C. To preserve the temperature integrity of the irrigation fluid, it was transported to the operating room immediately prior to the procedure’s timeout and only opened after anesthesia induction and patient draping were completed. This protocol was implemented to minimize thermal loss and ensure that the warmed solution remained at or near physiological temperature throughout the hysteroscopic intervention.

The TruClear™ system’s integrated fluid management display, was continuously recorded via video throughout each procedure. This allowed for precise post-procedural analysis of time intervals corresponding to specific fluid deficit thresholds. The time elapsed to reach fluid deficit volumes of 100 mL, 150 mL, 250 mL, and the total recorded fluid deficit was extracted from the video recordings to ensure accurate and unbiased measurement of fluid absorption rates during hysteroscopy.

Excluding the distention fluid temperature, all other steps during the procedure were as routinely used. Presurgical and post-surgical blood samples for sodium concentration and osmolality were collected. Women's pain perception was assessed an hour after the procedure using the Visual analog scale (VAS Scale).

Demographic and clinical characteristics were collected from the women's medical records. Operative and postoperative data were also gathered, including the indication for surgery, duration of the procedure, and any complications that occurred during or after the operation. These complications included uterine perforation, bleeding, fluid overload (including electrolyte disturbances, osmolality changes, pulmonary embolism, and pulmonary edema), as well as postoperative complications such as hemorrhage, endometritis, vascular events (thromboembolic events), and ileus.

The primary outcome was defined as the difference in median time to reach a fluid deficit of 250 mL between the temperature groups. The 250 mL threshold was selected as a clinically pragmatic intermediate value intended to be high enough to detect potential group differences while still reflecting the expected low fluid deficits in predominantly diagnostic hysteroscopic procedures. Secondary outcomes included differences in the total fluid deficit for the duration of the procedure, VAS pain scores, serum sodium concentration, and serum osmolality.

The study protocol was approved by the institutional Ethical Committee Review Board (ID HSC-MS-22–1004) on June 14, 2023 and was registered at the National Institutes of Health (NCT05622097).

### Statistical Analysis

#### Sample Size Calculation

Based on the literature, the deficit rate for hysteroscopic polypectomy is estimated 10 ml/min ± 2.7SD and for myomectomy 82 ml/min ± 27.2SD [10, 11]. Assuming that the room temperature would be associated to 30% reduction in the fluid deficit and in order to achieve an 80% power with a two-sided alpha of 0.05, 45 women were needed to be enrolled in each arm. 5 women were added to each group taking under consideration 10% drop down. Given the limited available data on time-to-threshold outcomes in hysteroscopy, published estimates of fluid deficit rates were used as a clinically relevant surrogate for sample size calculation, as both parameters reflect the same underlying process of intravasation and are directly proportional. Calculation of sample size was done using the Gpower software.

Analysis was done by intention to treat. Validity of the data was tested using the Shapiro–Wilk or Kolmogorov–Smirnov tests. Data are presented as median and interquartile range (IQR). Comparisons between groups were performed using the independent samples *t*-test or Mann–Whitney U test, as appropriate. The chi-square and Fisher's exact tests were used for comparison between categorical variables. A multivariable regression analysis was performed to learn the independent factors associated with the total deficit, including: age, BMI, fluid temperature, indication for surgery and tissue shaver use. Significance was accepted at p < 0.05. Statistical analyses were conducted using the IBM Statistical Package for the Social Sciences (IBM SPSS v.19; IBM Corporation Inc, Armonk, NY, USA).

## Results

**Fig. 1:**
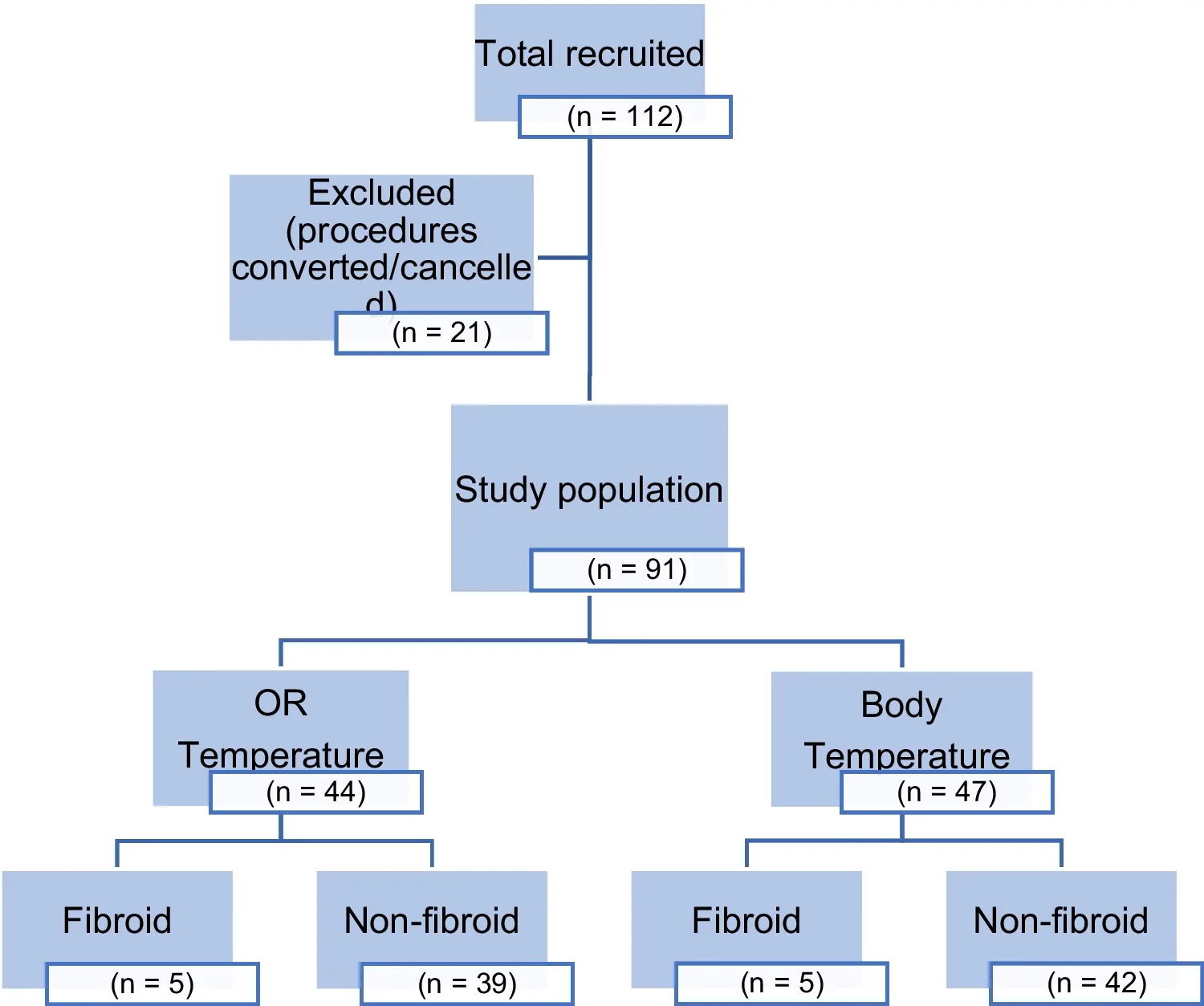
Study population

A total of 91 women met inclusion criteria, of them 44(48%) in the room temperature group and 47(52%) in the body temperature group (Fig. 1). Patients' demographic and clinical characteristics are summarized in Table 1. The median age and BMI were comparable between the groups. The study included participants from all ethnicities, with the largest being Afro-American, comprising one-quarter of the study population.

**Table 1:** Patient demographics and clinical characteristics

	OR temperature (*n* = 44)	Body temperature (*n* = 47)
Age (years)	38 (33–45)	39 (32–45)
BMI (kg/m2)	32 (27–40)	29 (27–37)
Gravidity	1 (0–3)	1 (1–3)
Parity	1 (0–3)	1 (0–2)
S/p CS	0	0
Ethnicity*		
White	7 (15.9)	4 (8.5)
African American	16 (36.4)	23 (48.9)
Asian	1 (2.3)	2 (4.3)
Hispanic	12 (27.3)	11 (23.4)
Other	5 (11.4)	3 (6.4)
Not specified	3 (6.8)	4 (8.)

Data are presented as median and IQR

^*^n, (%)

**Fig. 2:**
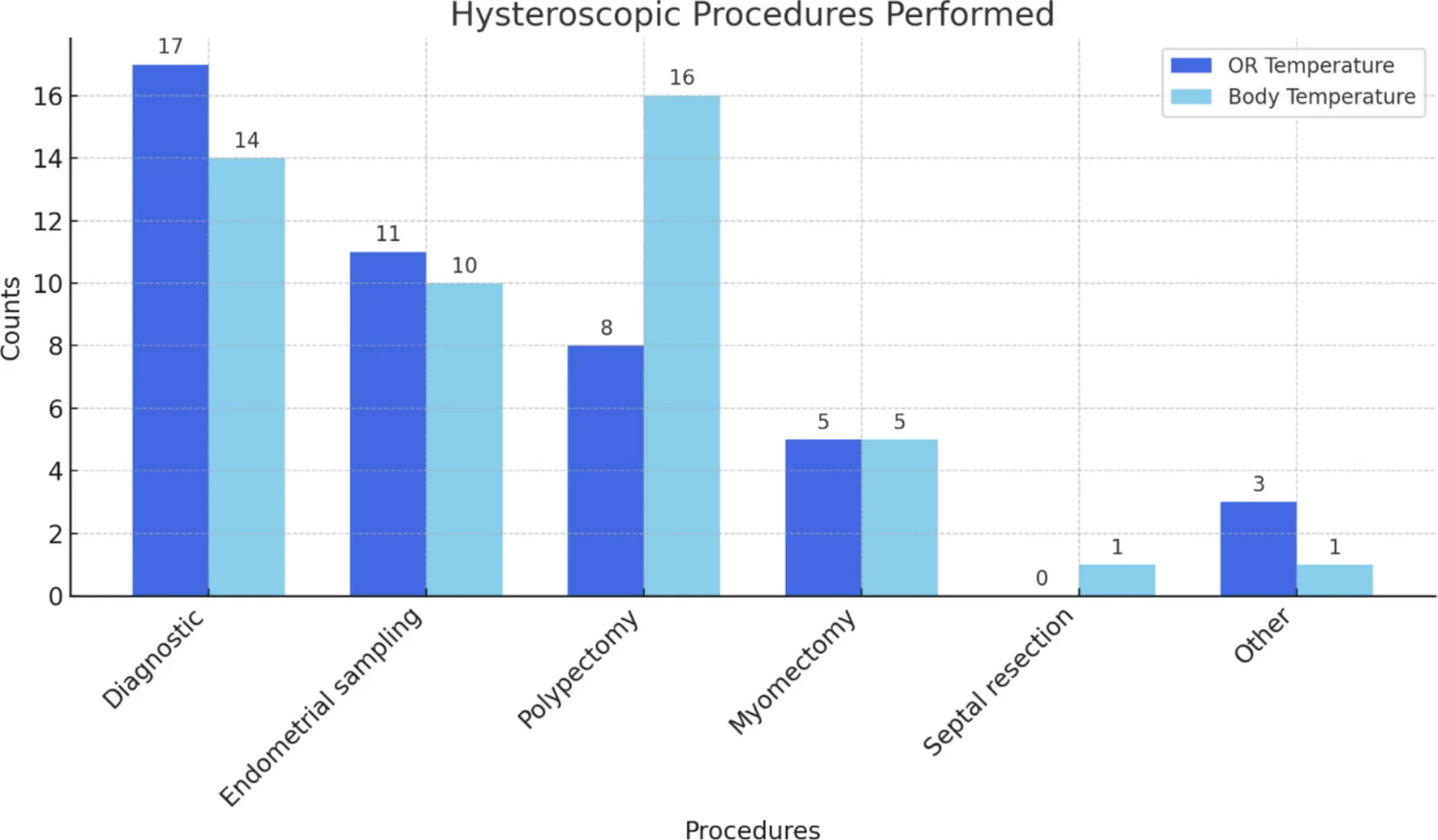
Hysteroscopic procedures

The most common procedure performed was diagnostic hysteroscopy (Fig. 2). Table 2 outlines the hysteroscopic characteristics. The Mini TruClear™ hysteroscope was used in 93% of procedures in the room temperature group and 91% in the body temperature group (*p* = 0.76). The tissue shaver was used in more than half of the procedures, with similar rates between the groups [25 (57%) vs. 31 (66%), respectively; *p* = 0.53]. Interestingly, the time required to reach fluid deficits of 100, 150, and 200 ml was shorter in the room temperature group compared to the body temperature group, although the differences did not reach statistical significance (*p* = 0.31, 0.21, 0.25, respectively). The total fluid deficit was higher, and the total time to complete the procedure was shorter in the body temperature group, as expected; however, these differences were not statistically significant (Table 2).

**Table 2:** Hysteroscopic characteristics

	OR temperature (*n* = 44)	Body temperature (*n* = 47)	*P*-value
Type of hysteroscopy (mini-Truclear)*	44 (93.2)	43 (91.5)	0.76
Use of tissue shaver*	25 (56.8)	31 (66)	0.53
Fibroids*	5 (11.4)	5 (10.6)	0.91
MAP (mmHg)	79 (72–87)	80 (73–92)	0.67
Time to 100 cc (sec)	52 (23–200)	60 (23–106)	0.31
Time to 150 cc (sec)	69 (23–215)	103 (38–241)	0.21
Time to 250 cc (sec)	155 (67–314)	219 (83–444)	0.25
Total time (sec)	405 (200–950)	360 (200–605)	0.53
Total deficit (ml)	658 (452–983)	720 (460–1194)	0.45
Pre-op sodium (mmol/L)	139 (137–140)	138 (137–139)	0.34
Pre-op osmolality (mOsm/kg H₂O)	289 (287–292)	287 (283–292)	0.15
VAS Score (1–10)	4 (0–6)	1 (0–6)	0.38

Data are presented as median (IQR)

*MAP* Mean arterial pressure, *VAS* Visual analog pain score

^*^ *n*, (%)

As hysteroscopic myomectomies are expected to expose more uterine vessels, potentially influenced by vasodilatation from the warmed solution, leading to increased intravascular absorption, an additional sub-analysis was performed based on the indication for surgery (Table 3). All deficits measured were comparable between the groups for both fibroid and non-fibroid procedures. The use of warm solution did not significantly influence the fluid deficit.

**Table 3:** Sub-analysis by indication for surgery

Time to deficit (sec)	OR temperature (*n* = 44)	Body temperature (*n* = 47)	*P*-value
Fibroids	*n* = 5	*n* = 5	
100 ml	58 (14–180)	89 (33–96)	0.77
150 ml	48 (11–48)	96 (38–272)	0.29
250 ml	112 (72–232)	154 (98–154)	0.48
Total deficit	836 (533–937)	636 (394–1068)	0.60
Non-fibroids	*n* = 39	*n* = 42	
100 ml	52 (23–200)	53 (23–125)	0.70
150 ml	70 (23–221)	110 (38–241)	0.32
250 ml	169 (58–363)	235 (65–430)	0.36
Total deficit	648 (443–1002)	781 (460–1198)	0.37

Data are presented as median (IQR)

Postoperative pain evaluation using the VAS score in the recovery room revealed lower scores in the body temperature group compared to the room temperature group [4 (0–6) vs. 1 (0–6); *p* = 0.38]. This trend is consistent with previous reports associating warm temperature with reduced post-procedural pain, although it did not reach statistical significance.

A multiple regression analysis was conducted to identify independent factors associated with the total fluid deficit, including age, BMI, fluid temperature, indication for surgery, and use of the tissue shaver. The use of the tissue shaver was found to be the only statistically significant independent factor associated with the fluid deficit [B = 396.19, 95% CI: 155.93–636.45, *p* = 0.002] (Supplement Table 1).

## Discussion

### Principle Findings

The main findings of the study are: 1- Fluid temperature did not significantly influence the fluid deficit during hysteroscopy; 2- Similar findings were observed in both fibroid- and non-fibroid–indicated hysteroscopies, though subgroup analyses were limited by small sample sizes in the fibroid cohort.; and 3- A trend toward lower VAS scores was observed with body-temperature fluid.

### Interpretation

The adoption of in-office hysteroscopy is influenced by both appropriate patient selection and the availability of necessary equipment and resources. Vaginoscopy is the preferred entry technique, as it has been shown to reduce intra-procedural and post-procedural pain [12].

Hysteroscopy is a minimally invasive procedure that can be performed either in an office setting or an operating room, depending on the underlying pathology [13]. Recently, an increasing number of clinics have started offering in-office hysteroscopic interventions, such as endometrial sampling, polypectomy, and submucosal myomectomy. Advances in technology have facilitated the use of smaller hysteroscopic equipment, eliminating the need for cervical dilatation, which is often associated with significant patient discomfort [14, 15]. Additionally, the adoption of vaginoscopy as a preparatory step has further minimized the potential for painful vaginal and cervical interventions, enhancing patient comfort during the procedure [16]. n this study, we included patients who underwent the procedure in the operating room to utilize the TruClear™ hysteroscope, which features a fluid collection system essential for the precise evaluation of fluid deficit. This equipment is exclusively available in the operating room at our institution. To enhance patient comfort, all procedures were initiated with vaginoscopy, minimizing the potential for pain or bleeding associated with the intervention.

The primary benefits of performing interventional hysteroscopy in an ambulatory setting include avoiding general anesthesia, reducing operating room costs, and saving time for the patient [17]. However, one of the main challenges is managing patient pain, which is often a limiting factor in completing the procedure [18, 19]. To address this issue, surgeons have introduced the use of warmed solutions, which have been shown to significantly reduce Visual Analog Scale (VAS) pain scores [8]. The pain-reducing effect of warmed solutions has been demonstrated previously in other procedures, including warmed humidified insufflation (WHI) in laparoscopic abdominal surgeries, hysterocontrast sonography, and hysterosalpingography [20–22]

The use of warm solution as a distension medium during hysteroscopy for pain alleviation remains controversial [8, 23–25]. Isaat et al. [23] conducted a prospective randomized placebo-controlled study involving 100 women undergoing office hysteroscopy. Visual Analog Scale (VAS) scores were compared between women treated with 0.9% normal saline fluid warmed to 36 °C and those treated with saline at room temperature (25°C). The study found no significant differences in VAS scores during the procedure (*p* = 0.554) or 5 min afterward (*p* = 0.12). In contrast, Gulucu et al. [8] assessed six VAS measurements during office hysteroscopy in 100 women randomly assigned to receive distension fluid at 37 °C or 25°C. They reported significantly lower VAS scores in the warm fluid group during specific phases of the procedure, including the cervical ostium transition, intra-cavity phase, and at the end of the procedure (*P* < 0.05).

A recent meta-analysis of five randomized controlled trials involving 441 patients concluded that using warm saline in office hysteroscopy effectively reduces pain during and after the procedure and improves patient satisfaction [9]. In our study, no statistically significant difference in VAS pain scores was observed between the two fluid temperature groups. However, there was a trend toward lower pain scores with the warm solution (4 vs. 1; *p* = 0.38).

Concerns regarding the use of warm solutions during hysteroscopy are primarily related to the potential for vascular expansion, which may increase intravascular absorption. This could elevate the fluid deficit during the procedure, limiting its duration and increasing the risk of hysteroscopy intravascular absorption (OHIA) syndrome [26]. Although rare, OHIA syndrome can cause significant morbidity and even mortality following what is generally considered a minor and safe procedure expected to conclude uneventfully.

Studies evaluating fluid deficit as a function of irrigation solution temperature during hysteroscopy are limited and largely conducted on simulation models. Fonseca et al. [4] investigated the role of solution temperature on absorption rates during endoscopic surgery. They assessed the time to reach fluid overload at various temperatures—17°C, 27 °C, and 37°C—using both electrolyte and non-electrolyte irrigation solutions. Their findings indicated that increasing the solution temperature from 17 °C to 37 °C resulted in a predicted 54% increase in the mean absorption rate and a 65% reduction in the theoretical "safe" duration of surgery for both solution types.

The findings of this study did not demonstrate a significant difference in fluid absorption based on the temperature of the fluid. Although a trend was observed in the warm solution group, where shorter durations were noted to reach specific fluid volumes (100, 150, 250 mL), the differences were not statistically significant. This trend was also apparent in cases where hysteroscopic myomectomies were performed, as more vessels are typically exposed, potentially increasing fluid absorption. However, this difference did not reach statistical significance in this subgroup either.

A possible explanation for this lack of significance could be the relatively short duration of the procedures, which were influenced by the nature of the pathology. The procedures conducted in this study reflect the variety typically performed in an outpatient clinic (Fig. 2), making these findings relevant. They may provide reassurance to surgeons regarding the use of warm solutions without the concern of potentially limiting procedure duration or increasing patient risk.

Another possible explanation is the smaller temperature differential between the groups in this study, which may not have been large enough to produce a significant difference in fluid deficit, as demonstrated by Fonseca et al. [4]. The chosen temperature ranges (body temperature and room temperature) were based on previous studies and clinical practices [8, 9]. Since no procedures were terminated due to significant fluid deficit, the selected temperature difference was likely sufficient for the evaluation and provides scientific support for the clinical use of both. However, further studies might investigate the impact of cooling distention fluids in procedures that require longer durations, such as larger myomectomies (FIGO 2–3), which are often performed in two stages due to fluid deficit limitations.

### Strength and Limitations

To the best of our knowledge, this is the first randomized controlled trial comparing hysteroscopic fluid deficit at room temperature versus body temperature in humans. All procedures were conducted in a single surgery center by a specialized and consistent surgical team, which minimized potential confounders that could have influenced the study results. Additionally, the external validity of the study is high, as it included a diverse population with various gynecological pathologies.

However, several limitations of this study should be acknowledged. First, the study sample was relatively small, with a limited number of participants treated for fibroids. Since fibroids are more likely to be associated with increased fluid absorption and may lead to early termination of the procedure, a larger cohort would have provided more robust and generalizable data. Second, fluid loss during the vaginoscopy portion of the procedure was not measured separately, potentially leading to an overestimation of the total fluid loss associated with the procedure. Third, all procedures were performed under general anesthesia, which prevented assessment of intraoperative pain related to fluid temperature and limits the applicability of the findings to office-based hysteroscopy, where patient comfort and tolerance are key considerations. Lastly, both soft and dense tissue shavers were included in the study. Although these devices share a similar mechanism of action, they differ in their application and efficiency—particularly in treating larger or more fibrotic lesions—which may have introduced variability in fluid absorption outcomes.

## Conclusions

The aim of this study was to evaluate the association between irrigation fluid temperature and fluid deficit during hysteroscopic procedures. The study findings suggest that irrigation fluid at body temperature, as compared to room temperature, does not significantly impact fluid deficit during hysteroscopy, regardless of the procedure’s indication. To the best of our knowledge, this is the only randomized controlled trial comparing the effects of room-temperature versus body-temperature irrigation fluid on fluid deficit during hysteroscopy in humans.

Despite concerns regarding the potential for higher fluid deficits with warm fluids, the findings support the use of body-temperature irrigation fluid, which may help reduce patient discomfort during ambulatory hysteroscopic procedures. This information is valuable for both physicians and patients when selecting optimal procedural conditions. However, these findings should be interpreted with caution given the relatively small sample size, the exploratory nature of subgroup analyses, and the fact that all procedures were performed under general anesthesia, which may limit applicability to office-based hysteroscopy settings. Larger studies are needed to validate these results and assess their generalizability across broader clinical scenarios. Future investigations should consider comparing multiple irrigation fluid temperatures—including cooled solutions—to more comprehensively evaluate their impact on fluid absorption, procedural efficiency, and patient-reported outcomes, particularly in longer procedures or those involving larger fibroids where fluid deficit may be clinically limiting.

## Supplementary Information

Below is the link to the electronic supplementary material.

Supplementary file1 (DOCX 14 KB)

## Data Availability

Data will be available upon request.
