# Supplementary material for: The Influence of Solution Temperature on Fluid Deficit During Hysteroscopy
Source: Reprod Sci. 2026 Jul 13;33(8):1556–63. doi: 10.1007/s43032-026-02151-2 (PMC13489988; doi:10.1007/s43032-026-02151-2)
Supplement: Supplementary file 1 [file 43032_2026_2151_MOESM1_ESM.docx]

**Supplement Table 1: Multi regression analysis for the total fluid deficit**

| **Factor** | **B** | **P value** | **95CI%** | |
| --- | --- | --- | --- | --- |
| Age | 1.09 | 0.78 | -6.69 | 8.88 |
| BMI | 0.86 | 0.85 | -8.24 | 9.97 |
| Temperature | 41.68 | 0.65 | -142.12 | 225.34 |
| Indication | 4.77 | 0.81 | -33.95 | 43.49 |
| Tissue shaver | 396.19 | 0.002 | 155.93 | 636.45 |
